# Selections and Abstracts

**Published:** 1914-07

**Authors:** 


### SELECTIONS AND ABSTRACTS

### THE CURE OF SYPHILIS BY COMBINED SALVARSAN-MERCURIAL TREATMENT.

By Professor W. Scholtz, Koenigsberg, Germany.

While the strikingly quick and reliable action of salvarsan on the clinical phenomena of syphilis must be concurred in by the opponents of salvarsan treatment, there still remains a lively discussion regarding the value of salvarsan treatment for the permanent cure of syphilis and the dangers involved in this treatment. As momentous and noteworthy as it is, however, to collect and publish all the material in reference to the results and harmfulness of treatment with salvarsan, the salvarsan debate has now, however, run into a false channel. The tendency has been to generalize the experiences obtained with salvarsan when given in a single definite form of application and dosage, and although I have already repeated and indicated before the International Dermatologic Congress in Rome the unjustness of such generalization, this unfortunately still continues to go on in an expansive manner, especially from the opponents of salvarsan treatment.

To-day the critical judgment of the value of salvarsan therapy no longer concerns itself with the question as to how far an unqualified application and dose of salvarsan can do harm and how frequently recurrences in syphilis can take place through an insufficient salvarsan treatment for example, after a single injection, but it concerns itself with this: WHAT CAN BE ATTAINED IN A JUDICIOUS FORM, WITHOUT DANGER TO THE PATIENT, BY SALVARSAN THERAPY OR SALVARSAN-MERCURIAL TREATMENT? In order to judge this we must not throw together in a collective statistic the results, or rather, the deficient results and unfortunate cases which have been utilized by others in various forms; but the critical examination of this question must be based on the reports and statistics of those authors who have obtained the best results in both directions, as well in regard to the permanency of action of the salvarsan as in regard to the dangers of it as a remedy or cure. The applied forms of treatment are thereby kept in check for corroboration by original directions and in this way the best form of application of the salvarsan may be conclusively established.

If it be shown that treatment with salvarsan in any form has not a permanency of action and that it is not sufficiently harmless, then salvarsan treatment or salvarsan-mercurial treatment must be abandoned as a standard method of syphilitic treatment and reserved only for certain cases. If it be shown, however, that there are methods of salvarsan treatment which not only act momentarily on the clinical phenomena in the well-known prompt manner but that they also have a permanency of action, which means, that they can cure syphilis definitely and that without any so-called danger to the patient, then these methods may be accepted as universal methods for the teatment of syphilis.

I hope to convince the reader through my following deductions and statistical communications that there already is such a method. In the DEUTSCHE MEDIZINISCHE WOCHENSCHRIFT and in the WOCHENSCHRIFT FUR PRAKTISCHE DERMATOLOGIE and before the Congress in Rome, I have repeatedly and distinctly pointed out the permanency of action of salvarsan-mercurial treatment in the form developed by us and finally on the ground of our results that took place for more than a year in which cases there was a CURE OF THE SYPHILIS by a single salvarsan-mercury KUR.*

The method of our treatment is, in short, as follows: In the beginning of the KUR we first of all give on two consecutive days, two salvarsan injections on each day in average small doses (ordinarily two of 0.25-0.3 and two of 0.2 and altogether 0.85-1.0 of salvarsan through the four injections). Usually we give the first injection of 0.3 salvarsan in the morning then at noon, after an interval of about four hours, the second injection of 0.2 salvarsan; on the next day again in the morning at most 0.25 salvarsan and at noon-time, 0.2 salvarsan as the last injection. We employ, almost exclusively old salvarsan, not because neosalvarsan is slightly less active, but because of its rapid dissociation and we consider it somewhat toxic and therefore dangerous than old salvarsan.

We inject the salvarsan in this form in several successive small doses in order to obtain a durable action of the medicament. As it is known, salvarsan when injected intravenously in relatively large doses will disappear from the blood after about three hours and consequently a prolonged action is possible only by several successive injections at intervals of some hours. That only absolutely fresh distilled water is employed in the preparation of the salvarsan solution and salt solution is, of course, self-understood.

Under these circumstances in fresh primary infections and in most cases of latent lues any reactions now tend to be absent. Only in fresh exanthemata or in old primary infections with multiple glandular swellings does a quick transitory rise in temperature, after the first injection and until after the second salvarsan injection, take place to 39 degrees (102.2°), seldom higher. It is therefore advisable in extensive exanthemata or in old primary infections with severe glandular swelling, and above all, if any prodromal symptoms exist, to begin with smaller doses than 0.2 (in strong prodromata, only

0.1 or 0.15) and at the second and third injections to use somewhat larger doses; or else instead of four injections to perform five; or in such cases the salvarsan KUR may be preceded by one or two mercurial injections with Hydr. sal., or 40 per cent. calomel; or else six to eight mercurial inunctions may be given. Immediately upon conclusion of the salvarsan treatment, usually at the end of the second salvarsan day, we begin with a mecury KUR and, of course, we mostly start with inunctions. Between the single inunctions we push with all vigor mercurial. injections with Hydr. sal., or even better, 40 per cent. calomel (pro injection 0.08 Hydr. sal., or 0.05 to 0.06 calomel). We also employ mercinol frequently, a 40 per cent. mercury oil, for injection although never by itself but always alternately with Hydr. sal., because mercinol is absorbed only very slowly and hence may easily produce a cumulative action.

Inunction KURS we carry out in this way: On each of four successive days we rub in one quarter of the body four or five grams of mercury resorcin. The first day one arm and one side of the trunk of the body are rubbed; on the following day the similar portion of the body on the other side is rubbed; then follows one leg, hip and buttock and lastly, the other leg, etc., in a similar manner. On the fifth day the patient rests and on the sixth day he is given a bath and receives mercurial injection. After the injection there is an interval of one or two days and then follows another bath after which the inunctions are repeated and at the end of five inunction days another injection is made as before.

We employ usually this last form of combined injection-inunction KUR because from our experiences it is better tolerated relatively and we can also give relatively more mercury to the patient and prevent strong local and general reactions.

After the salvarsan injections I order the patient to be packed warmly in bed so that he will perspire freely and in this way, especially in the skin, the salvarsan circulates more actively. After each injection of salvarsan the urine should be examined for albumin and the quantity of urine also measured so as to establish the functionating power of the kidneys. By employing the concentrated injections of neosalvarsan the KUR in this form may also be carried out in the home of the patient very conveniently and easily. To this end 5-10 c. c. of water are boiled in a test-tube, allowed to cool and 0.3 or 0.4 neosalvarsan dissolved therein. This solution is then immediately injected intravenously. Every practicing physician who has mastered the technic of intravenous injections is able to carry through this procedure very easily. To be sure, the solution must flow faultlessly into the vein and not a drop should escape into the surrounding tissue otherwise thrombosis and necrosis may occur.

We ourselves, as indicated above, still employ old salvarsan dissolved in a larger fluid medium (0.1 salvarsan in 50-60 c. c. of 0.6 per cent. sodium chlorid solution) chiefly because we believe that with the large amount of fluid there is a preliminary "washing out" of the tissue for the penetration of the salvarsan into the tissue, which is perhaps not without some significance.

In extensive exanthemata the injection KUR has, undoubtedly, a special value by reason of the local action of the mercury on the poisons in the skin and through absorption from the skin it also exercises a strong action on the glands. Furthermore it is possible that the special form in which the mercury is taken up by the skin from the inunction KUR (inhalation of the vapor through the lungs!) and the surrounding of the body by a mercurial atmosphere, effective action is obtained. Also injections have on account of their continuous absorption into the system and through the manner in which the mercury circulates, their special action and it is quite plausible that by a combination of both these methods a more favorable action on the disease is obtained than through the action of these methods alone. From which it follows that in spite of the very large quantities of mercury used we have obtained less and lighter mercurial intoxication or stomatis from the mixed injection-inunction KUR than by injections or inunctions alone, because we have better control of the administration of mercury. By inunctions the danger from stomatitis is reduced by injections, the danger from a general intoxication is guarded against.

In this form we carry the mercurial KUR through for four weeks, then follows the second salvarsan KUR which is carried out essentially just as the first was but in weaker patients it may be reduced in strength. On the first day, for example, we give only 0.25 and 0.2 salvarsan; on the second day twice 0.2; or we can give three injections of 0.25, 0.3, then 0.2 and on the second day again 0.3 of salvarsan. At the end of the second salvarsan KUR is again begun and carried through for from fourteen days to three weeks in a similar way as the first time.

The intensity of the second salvarsan KUR as of the further mercury KUR depends essentially upon the RESULTS OF BLOOD EXAMINATION WHICH IS CONVENIENTLY CARRIED OUT AT THE SECOND SALVARSAN KUR. Even if the result be weakly positive the salvarsan KUR is gone through with the same intensity as the first time and the mercury is then continued for fully three weeks with an equal strength as at the first time. If the reaction is still perceptible at the end of the second salvarsan KUR or by the completion of the second mercury KUR then a third salvarsan treatment of at least one day is given. In such an event two injections are administered of 0.3 and 0.2 salvarsan. As a rule, though, in primary and secondary lues at the time of the second salvarsan treatment, the Wassermann reaction is fully negative so that naturally a further continuation of the KUR would be unnecessary.

That the method of procedure of the salvarsan KUR and of the mercury KUR must be adapted, naturally, to the constitution of the patient, according to his weight, etc., with small deviations, and especially as regards dosage goes without saying.

The salvarsan-mercury KUR performed in the manner described makes certain demands upon the organism and the patients usually become somewhat weakened and tend to lose weight. The patient must therefore conserve their health.

All bodily and mental exertion and above all, unnecessary amusements, night life, alcohol, etc., must be shunned. The skill of the physician comes into play, especially in cases where stomatitis or other evil effect have entered as a disturbing factor, in the proper arrangement of days of rest and means of nursing in order to provide against more actual harm to the organism. An exact control by the urine, a control by the weight, by the mouth and the most scrupulous attention to the mouth are, naturally, indispensable. For rinsing the mouth I always use a solution of perhydrol (sol. perhydroli 10-400, a teaspoonful of which is diluted with ½ glass of water) and this may also be painted on the gums with the undiluted solution, in stomatitis, morning and night. Moreover, these patients have a tendency, after the course of treatment, to improve wonderfully whereas during the KUR they had lost from 10 to 15 pounds in weight.

The results that we have obtained from this form of salvarsan-mercurial treatment are exceptionally good and especially in primary and fresh secondary lues one or two years after infection, particularly brilliant, as from our following statistics.

A year ago all the cases that went through the regular course of treatment were examined clinically and serologically, the findings collected and the statistics published in the DEUTSCHE MEDIZINISCHE WOCHENSCHRIFT, No. 30, 1913. In order to obtain correct percentages we included in the statistics only those patients whom we were able to control clinically and serologically from three to six months. The cases in which evidences continued to be present remained, as a rule, about two or three months under our observation and these were also admitted to the statistics when they could not be followed up further. The cases free from all traces of infection which were under observation for an equal period of time were also made use of in the statistics. The total number of cases of primary and fresh secondary lues (first and second year after infection) on which we could rely upon was 316. One part of these cases we had during the past year under further observation and serological control so that the statistics at the present time have an added value. The number of cases under further observation which previously remained but from three to six months under observation is still relatively small (about 15 per cent. of the total). About two-thirds are cases which were under observation for from one to two years and in about 10 per cent. the period of observation was from two to three and one-half years.

Altogether in the 316 cases there were relapses in 28 cases clinically. In 13 cases there was only a positive Wassermann as a relapse; in some of them this had never become negative. In round numbers there were 13 per cent. recurrences, of which 9 per cent. were clinical and 4 per cent. serological.

Of PRIMARY lues we had 85 patients sufficiently long under observation and of these there were 6 relapses (7.5 per cent.).

Of SECONDARY lues within the first two years after infection we observed 35 relapses among 231 patients (15.5 per cent.).

There is quite a considerable difference between results in the clinic and in private practice, so that it is well to recapitulate the percentages in separate columns.

Of primary lues in private practice, 44 patients were treated, ALL OF THEM WITHOUT ANY RELAPSES. Of these, 3 were observed and serologically controlled only 3-6 months; 7 were observed and serologically controlled only 6-12 months; 25 were observed and serologically controlled only 1-2 years; 7 were observed and serologically controlled only 2-3½ years.

Of secondary lues in private practice there were 109 patients sufficiently long observed and of these 11 showed later recurrences, especially in the form of positive Wassermann reaction. In those free from residues the period of observation of the patients were:

18 from 3- 6 months.
28 from 6-12 months.
48 from 1- 2 years.
10 from 2- 3½ years.

In private practice, then, in fresh secondary lues there were only about 10 per cent. relapses, in primary lues 100 per cent. cures, in primary and secondary lues together 6.4 per cent. relapses.

In the clinic, on the contrary, in the same period of observation of 163 cases of primary and secondary lues, there were 30 relapses, especially positive Wassermann, a round 18 per cent.

That the results in private practice were considerably superior to those in the clinic is due to the fact that the private patients were more regularly examined clinically and serologically than the patients in the clinic, and also the patients in private practice had their entire treatment more carefully and intensively carried through than the clinic patients had. The latter not only performed the inunctions carelessly, but frequently also they came for the mercury injections very irregularly and allowed too long an interval between the first and second KUR to elapse. Also in the clinic the number of salvarsan injections was more frequently restricted than in private practice, in each KUR by two or three injections.

Perhaps the less favorable results in the clinic are only apparent and to be attributed to the fact that clinic patients when they notice no symptoms of the disease and feel as if they were cured do not come nearly as regularly for the control examination as do private patients. It is therefore possible, and from our clinic material it seems to me probable, that the patients who sustained relapses have on these grounds placed themselves as unexceptions whereas the percentage of cured patients who allowed themselves to be regularly examined, to the total of those cured is even relatively smaller.

The probability of cure with the correct carrying through of the course of treatment in primary lues is 95-100 per cent., and after one year's control the probability of later relapses in primary lues should be zero.

In secondary lues the probability of cure under correct treatment is fully 85 per cent., and after one-half years control and exemption from syphilitic symptoms a relapse is extremely improbable; of these fully 95 per cent. show probability of ture whereas those cases under control for one year show about

1 per cent. and at the highest 2 per cent. of recurrences (*i. e.*, about 98 per cent. of cure).

The marriage census, especially in secondary syphilis, after one or two years' control, we will not yet give, for while the probability of a relapse is exceptionally slight, any residue, as can be foreseen, would consequently be strongly contagious. On these grounds, I would desire a further control, as a rule, of two or three years before making a communication as to the marriage census. After three years, I believe, we can give it with far more correctness and far more assurance than before.

Another question that comes up, especially in our case, if the cases that have shown themselves as being healthy clinically and seologically for one, two or three years may be spoken of as CURED, or if we must reckon fith the possibility that in spite of this freedom of the first two or three years, tertiary or so-called metaluetic affections will later make their appearance. I believe that there is no ground for such scepticism and more so that experiences with the clinical course of syphilis in such cases allows an expectation of an actual definite cure.

To be sure, syphilis may have a chronic course and may after ten or twenty years, lead to phenomena of disease; no clinical indications of the disease will be demonstrable on the skin particularly, years afterwards. But in the first place, from our present experiences the blood of such patients during the whole terminal so-called latent period is strongly positive to the reaction of Wassermann and in the second place if the COURSE OF SYPHILIS, AT LEAST IN THE FIRST YEARS AFTER INFECTION, IS AN EXTRAORDINARY TYPICAL AND REGULAR ONE, its course in isolated cases in the later period may be exceptionally irregular. For the typical blood reaction makes its appearance with the greatest regularity seven or eight weeks after infection and remains without treatment—aside from the indistinct, transitory fluctuations—positive during the next year in the greatest number of cases. According to our present experiences, in cases showing late phenomena, this reaction remains as a rule lastingly positive during the whole "latent" course.

Under the MERCURIAL course of treatment in primary and fresh secondary lues the clinical symptoms disappear and the Wassermann reaction becomes negative; but very seldom this reaction may, several months after completion of the course of treatment or at latest from one-half to one year, again become positive. As it is after mercurial treatment so is it after treatment with salvarsan. The frequent recurrences after incomplete treatment with SALVARSAN AND MERCURY, and also the rarer disappointments after radical carrying through of the salvarsan-mercurial treatment have shown this to be the case. In these cases the blood again becomes positive to the Wassermann in about from three to six months or at latest from six to twelve months after completion of the cure. A recurrent positive reaction after more than one year is a very rare thing which need not be considered further.

WE SEE AGAIN THEREFORE, THAT THE RECURRENCES FROM FAULTY SALVARSAN-MERCURIAL TREATMENT TEND TO MAKE THEIR APPEARANCE IN THE SAME WAY AND IN THE SAME TIME AS AFTER STRONG, SIMPLE MERCURIAL TREATMENT, so that we have not the slightest right to the view that treatment with salvarsan changes and delays the whole course of syphilis.

WHEN IN A DISEASE, THE TYPICAL SYMPTOMS WHICH APPEAR WITH ABSOLUTE REGULARITY IN A FEW WEEKS AFTER INFECTION REAPPEAR WITH SIMILAR REGULARITY WITHIN A FEW MONTHS AFTER A TREATMENT THAT IS ESSENTIALLY SYMPTOMATIC IN ITS ACTION BUT WHICH REMAIN AWAY AFTER THE TAKING OF A DEFINITE COURSE OF TREATMENT, THEN, WE MUST CONCLUDE THAT THE DISEASE IS CURED.

A great part of the cases of syphilis in their presentation have so impressed us with their "latency" that we no longer venture any confidence in its course not even when no symptoms whatever are present. But after all from what we now know about the course of syphilis at least in its early period we know that at this time there is no durable latency. All possible symptoms in the skin may remain away but the Wassermann reaction becomes established with the greatest regularity and even after a merely symptomatic acting course of treatment it reappears after several months. When, therefore, after a qualified salvarsan-mercurial treatment all symptoms of disease, as well as a positive Wassermann reaction, have remained absent for years in primary lues in about 95 per cent. of cases, in secondary lues 85 per cent. of cases (reappearing again as exactly after a mercurial course of treatment in 5 per cent. and 15 per cent. respectively at the same time and in the same form) then the only logical conclusion we can reach is that in the first series a definite cure has been attained through the course of treatment whereas in the second series of cases the course of treatment is disappointing.

It is more difficult to reach an opinion relative to the definite effective results of salvasan-mercurial courses of treatment in later stages because we have not yet sufficient knowledge regarding the course of the blood reaction as well as the time of reappearance of the reaction after a course of treatment.

With our course of treatment two-thirds of the cases in later stages show a negative reaction and later recurrence of the reaction is very seldom observed. In this respect our experiences agree with those of Gennerich.

In the sense of a cure by the treatment of syphilis in the early stages with the combined salvarsan-mercurial treatment this observation speaks further: we have treated seven diseased children who showed from ¾-1½ years after the KUR (*i. e.*, 1½-2½ years after birth) no symptoms whatever of syphilis. In two instancesthe parents were syphilitic. Examination of the blood of the children gave complete negative results in addition to no clinical evidences whatever, after treatment. Finally three other observations point in a similar way; which must be viewed as reinfections These cases have to do with typical primary infections with more or less general glandular swelling. There was a TYPICAL INCUBATION PERIOD after coitus with a prostitute and yet in the beginning there was a negative or a very weakly positive blood reaction.

We hold the standpoint that with the salvarsan-mercurial treatment in the form employed by us, a cure is attained in 95

per cent. to 100 per cent. of cases of primary syphilis and in 85 per cent. of cases of secondary syphilis, and that this cure is definite. In our judgment we do not stand alone; particularly Gennerick reported in the MUNCHENER MEDIZINISCHE WOCHENSCHRIFT, results similar to ours.—Urologic Cutaneous Review.

*Translator's Note.—It must be remembered that the German word "kur" is not identical with the English word "cure." In recent years it has found its way into English usage because of its brevity. "Kur" simply means a course of treatment regardless whether a "cure" has resulted therefrom or not.

---

### HOOKWORM DISEASE, ITS RAVAGES, PREVENTION AND CURE.

By JOHN A. FERRELL, B. S., M. D., WASHINGTON, D. C.
Assistant Administrative Secretary, Rockefeller Sanitary Commission.

Hookworm in sections in which it prevails is one of the most common, most insidiously harmful, and most easily prevented diseases known to man. It causes human suffering and economic waste altogether out of proportion to its apparent death-rate. Many ills which have been attributed to mental and moral weakness of whole bodies of people are now definitely known to be due to this infection, and curable with its cure. Its eradication is one of the most important and pressing problems before the people of the southern half of the United States and of other semi-tropical and tropical lands. Moreover, the progress which has been made in recent years completely demonstrates at once the vast benefit, in terms both of human happiness and of industrial efficiency, attendant on

*This paper is one of a series on Public Health, prepared at the request of the Council on Health and Public Instruction, and reprinted for distribution to the public through state boards of health and other agencies. Other articles are being prepared and will be issued later. A list of the pamphlets can be secured from the Council on Health and Public Instruction, American Medical Association, 535 North Dearborn Street, Chicago.

stamping out of the disease, and also the complete adequacy, in stamping it out, of perfectly simple precautions by the primary rules of health and even of common decency.

### Where Hookworm Disease Is Found.

Hookworm disease is found more or less prevalent in the entire zone lying within 30 degrees north and 30 degrees south of the equator, or in practically all the tropical and semi-tropical countries, in which are more than half the people of the globe. In many of these countries this anemia-producing infection is extremely prevalent, in a severe form. In Porto Rico and the low-lying district of Colombia, and in many of the sugar plantations of Ceylon and Dutch Guiana, for example, infection was found to involve 90 per cent. of the entire population. Since 1904 more than 300,000 persons have been treated for hookworm disease in Porto Rico, with the result that, whereas in 1900 and 1901 the deaths from anemia numbered 11,875 or 33.2 per cent. of the total deaths in the island, and in 1902 and 1903, after some treatment for hookworm, 6,830; they had fallen in 1904 and 1905 to 4,693, and continued to decrease until they were in 1906 and 1907 only 1,134, and in 1907 and 1908, 1,785. The majority of Porto Ricans, long noted for laziness and shiftlessness because of this anemia, are now known to have been the victims of a specific infectious disease; the wholesale treatment of which is revolutionizing the island, and bringing health and prosperity where almost universal misery and poverty reigned.

In the United States, the disease is found thoughout the states south of the Potomac and Ohio rivers, in Arkansas, Missouri, Oklahoma, and Texas, and also in California. Its prevalence and severity vary widely within the state and even in a county, in some localities less than 1 per cent. of the people being infected, and in others more than 90 per cent. Generally speaking, the heaviest infection is found on the light, sandy soil of the coastal plains, the lightest infection on the stiff, clay soil of the Piedmont region, and an intermediate infection among the foothills and mountains. It is particularly a disease of the agricultural district, which goes far to explain the long-puzzling lack of physical and intellectual vigor to be noted among large classes of people in what ought to be one of the healthiest and most prosperous sections of the country. Examination of more than 415,000 school children during the four years 1910-1913 in 413 counties of the eleven Southern states has revealed 43 per cent. of them infected. Of more than 700,000 persons of all ages taken at random in the same territory, 35 per cent. were found to be suffering from this disease, and in a vast majority of cases were completely cured.

When individual treatment has been accompanied by simple sanitary reforms to prevent reinfection, most noteworthy benefits have been derived by entire communities. For example, in the camps of the Continental Coal Corporation, at Pineville, Ky., where 1,800 men were on the company's pay-rolls, in June, 1911, about 65 per cent. were infected with hookworm, there were about 150 cases of typhoid, and cases of bowel complaint were numerous. Soil pollution through lack of sanitary water-closets was practically universal, the water-supply was contaminated, and flies had free range. Measures were taken to eradicate the hookworm by providing sanitary closets, which stopped soil pollution and prevented flies from carrying the infection back into the houses, and the water supply was also safeguaded. As a result, in the year following, the same force of men loaded over 33 per cent. more coal on the cars than they did in the year before. Also there was not a single case of typhoid in the camps during the summer of 1912, and cases of diarrhea were reduced to about one-half.

### How the Hookworm Affects Its Victims.

For generations hookworm disease has been insidiously spreading unrecognized and unchecked over the countries of the globe having a mild climate. Its victims, numbering many millions, have through centuries, no doubt, been hosts to the small blood-sucking intestinal parasite which causes the disease. Their strength has been sapped, their vitality lowered, their physical and intellectual growth stunted. They have been mastered in war, commerce and industry by the more hearty people of colder latitudes to the north. The social and economical importance of the disease is therefore almost beyond comprehension. The infection is in most instances so insidiously acquired by the unsuspecting victim that he and the members of his family, who are probably likewise being infected, do not know just when the effects of the disease began to manifest themselves. In the course of a few summers, however, a once healthy family has become pale and puny; a once industrious family has become languid and backward in its work; a once prosperous family has fallen into debt; a once proud family, owning valuable property, has been reduced by an easily curable and preventable disease to tenancy and poverty. The children, once bright and well advanced in their school classes, begin to lose their zeal and their mental alertness when gradually robbed of their vitality. They fall behind in the struggle with their healthier classmates, and, finally discouraged and perhaps abused, give up school work in despair.

### Great Economic Loss Due to the Hookworm.

This is no exaggerated picture of the harm wrought by the disease. The economic loss already stated in the case of the Kentucky coal-miners is to be found wherever the disease prevails. The physically sound coffee-picker in Porto Rico picks from 500 to 600 measures of coffee a day. Dr. Wickliffe Rose reports that he found scores of infected men there who could pick only from 100 to 250 measures a day, and the manager of one of the great haciendas, or plantations, told him that the disease had reduced the average efficiency of labor on the coffee estates to from 35 to 50 per cent. of normal. One of the largest cocoa plantations in Ecuador reports that 300 laborers on the place were so reduced by the anemias of hookworm and malaria as to be able to do not more than one-third of a reasonable day's work. The manager of a large British Guinea sugar estate reports that treatment for hookworm has doubled the working power of the gangs. A single California mine employing over 300 men is estimated to have lost 20 per cent. of the wages paid, or $20,000 a year, because it had to carry on the pay-roll a large body of men to replace those periodically unable to work because of hookworm anemia. In the South Atlantic and Gulf States the infection is heaver than in California, and the loss on the farms and in the cotton factories is enormous. The weavers in the Southern mills are not naturally inferior to the European immigrants who operate the New England looms. Their labor is less efficient in some instances because vast numbers of them are victims of the hookworm. The Southern farms are not lacking in fertility, nor are their owners, whose fathers fought under Lee and Jackson, lacking in sterling virtues; but thousands of men and women with the best blood of the land in their veins are made improvident and slothful by this infection, and the hundreds of cases in which these supposed faults of character have been reformed by hookworm treatment prove beyond question the enormous moral and economic cost of the disease.

### Growth of Children Retarded.

Hookworm retards the development of children to a remarkable degree. School and college records show that infected students, even though not apparently ill, average lower in their studies than those found free from infection. In one woman's college the average standing of fifty-six girls found infected was 77.75 per cent., whereas fifty-six girls taken at random from those in the institution found free from infection averaged 89.28 per cent. Similarly in an academy, a group of twenty-five infected men and boys averaged 64 per cent., and a non-infected group beside them averaged 86 per cent. Teachers in all parts of the South report marked improvement in zeal and intelligence, as well as in weight and physical appearance of children immediately on being freed from the parasites. One case from the field director of the hookworm campaign in Prentiss County, Miss., may be given as fairly typical of the experience of physicians in this work.

"A young man that we treated furnished us the best recommendation that we could have had. He came to the office about the third week looking as if he had an advanced case of tuberculosis, and with a cough that appeared serious. He was such a picture of dejection, misery and lost hope, that he attracted the attention of the whole courthouse crowd and many of them watched eagerly to see the results of the examination. One man asked me after he left, if I could cure him, and I replied that we would do our best. This man then kindly stated that he thought that I was doing the boy an actual wrong by holding out hope, as it was his belief that the boy would die in a very short time. This boy was instructed to return every week for his treatment until informed that he was cured. He did; and it was a standing request by the court-house officials that they be called each week as W. L. came in, so that they could see for themselves whether he was improving or not. On his first trip, one week later, he came in, and there was evidence of much improvement. He came in with a smile, showing more interest in things around, telling jokes to fellows in the office, when just one week before he had stood listless without a word to any one except to answer 'yes' or 'no' when a question was put to him. He informed us that on this trip, although he had taken his medicine only a week ago, he was feeling a great deal better; and when placed on the scales, I found that he had gained a number of pounds in weight. He was given three treatments, one week apart, and after waiting two weeks following the third treatment, he was found free from infection, and weighed 18 pounds more than he did previous to the first treatment. On the day that he first appeared at the dispensary he could hardly walk, and the last time that I saw him he started with another boy to walk 7 miles to his home."

### The Penalty of Ignorance and Neglect.

Such facts, of which hundreds of the same import might be cited, show the heavy price which the people of the South have been paying in loss of money, health and intellectual power, for ignorance and carelessness concerning a disease which is easily diagnosed, easily cured and easily prevented. It is due solely to neglect of sanitation which permits the eggs of the small intestinal worms which pass with the excreta from the bowels of infected persons to find lodgment on the soil, and then hatch into infective larvae ready to carry the disease to some other human being or reinforce the infection of the original victims.

There is no other disease perhaps which is so well understood in every detail, and which can be so satisfactorily explained to a layman. Nor is there any other widely prevalent disease against which the lay community can so readily and surely protect itself by simple precautions. Its conquest virtually resolves itself into a problem of education against soil pollution.

The largest possible public therefore should be brought to know in detail the history of hookworm disease; the distribution and life cycle of the parasite causing it; its symptoms, method of cure, and, most important of all, means of absolute prevention.

### How Hookworm Disease Was Found.

Hookworm disease is not new. It is only newly understood. The symptoms of the disease were described in the records of the Egyptian Empire, but its cause was not known. The hookworm itself was discovered in 1838 by Dr. Angelo Dubini, an Italian, who while making a necropsy found the small white worm with its head buried in the membrane of the intestine; but that this worm had anything to do with anemia was not suggested. When, however, in 1877, Grassi, another Italian physician, identified the eggs of this worm in the feces of anemic patients, it was suspected that the parasite was the cause of the disease. About the same time, Dr. Colomatti, studying an obscure disease which had caused the death of many workmen on the St. Gothard Tunnel, discovered in the intestine of one of the tunnel victims more than fifteen hundred hookworms. This parasite, known as the Old World hookworm and named ANKYLOSTOMA DUODENALE, was carefully studied, its responsibility for certain types of anemia in Southern Europe demonstrated.

The prevalence of hookworm disease in the New World was not recognized till much later. Nov. 24, 1899, Major Bailey K. Ashford of the United States Army Medical Corps, while treating anemia supposedly due to starvation caused by the hurricane in Porto Rico, identified the hookworm as the real cause of the wide-spread disease. He, however, supposed the parasite found by him to be the Old World type. In 1902 Dr. C. W. Stiles of the United States Public Health Service, having found the same disease in the Southern States, identified its cause as a different species of worm, now known as the New World type, or NECATOR AMERICANUS. It was then discovered that the worm found in Porto Rico was of this species.

Subsequent discovery of the same worm as the cause of anemia among the victims of the African lowlands suggests that the so-called New World type was brought to Porto Rico and the Southern states by the slave trade. This form also prevails in India, and has been carried to Jamaica, Trinidad and British Guiana by the Hindoo coolies brought there as laborers. The disease caused by one or the other of the two types of hookworm is now known in practically all tropical or semitropical countries, but all the worms look alike to everybody except the expert zoologists; they produce practically the same symptoms; substantially the same treatment is effective with all, and they can all be prevented by the same means.

### HOW THE HOOKWORM LIVES AND WORKS.

The hookworm in adult life is a small, round intestinal parasite about ½ inch in length, and about the size of No. 30 sewing thread. As a type it is found to infect man and numerous animals, such as the dog, the fox and the cow; but the particular species infecting man have not been found in animals, nor those of the lower animals in man. Only the type infecting man will be discussed here.

The life of the hookworm is made up of two periods. During the first period the worms are microscopic in size and live in the soil. It is in the soil that they hatch from microscopic eggs which were deposited there with the excrement from some person having hookworm disease. Neither the eggs nor the larval worms hatching from the eggs can be seen with the unaided eye. These minute worms will live for perhaps ten or twelve months under favorable conditions of warmth and moisture; but they cannot develop beyond this point unless they gain entrance into the body of some human being and find their way into the intestinal canal, usually the upper portion of the small intestine. The drying heat of the sun and the freezing weather of winter are destructive both of the development of the hookworm eggs and to the life of the larvae. For this reason the disease is rare in deserts and in countries having protracted cold weather. A porous, sandy loam soil, having reasonable shade, affords the most favored habitat for the hookworm larvae. Though in stiff clay soil conditions are much less favorable to these little worms, we often find that enough of them have lived and entered into the second period of their life's existence to cause heavy infection.

The second period of the hookworm's life is spent within the body of a human being. The great majority of the worms never reach this stage in their development. In fact, many of the eggs never hatch. Yet when we stop to consider that in some localities 90 per cent. of the people are severely infected, and that each infected person will cast off with the excerta from one to four million eggs on the soil, we can appreciate than even with this loss it will be almost impossible for one to escape infection in a section where wastes from the human body are not properly disposed of.

With these facts before us, we at once want to know how these invisible filth-borne pests find their way to our small intestines. The answer may be a short story, but generally it is a long one.

It may be short because the little worms may cling to food which is swallowed by the unsuspecting victim. They may be carried on soiled hands; or, perhaps more frequently, may be swallowed along with uncooked foods, such as strawberries, plums, celery and lettuce, grown in or left to lie on ground polluted by human excreta.

Though some infection is thus taken through the mouth, the most of it is acquired through the skin in a very interesting way. In brief, this is the story of the hookworm's most common progress: Within a week of the hatching of the larvae they molt and shed their skin twice. They are protected by the second sheath, and are very tough. In this stage they possess the wonderful ability to burrow in a few minutes through the skin and to enter the tiny blood capillaries, where, carried along by the blood-current, they make a long journey through the body to the heart and to the lungs. In the lungs the blood-vessels are too small to permit them to pass through, so they begin to burrow again, and soon find themselves in the air-spaces of the lungs. Crawling along these they reach the wind-pipe and then the throat. Once there the worms are swallowed with saliva and food, and thus by this long journey find their way to the small intestine, where they fasten on its walls and begin their blood-sucking.

One would think that the hookworms would have great difficulty in penetrating the skin and the blood-vessels in the lung, yet they do it. The fact can be easily demonstrated by experiment. If the polluted soil in which the larvae are known to be is moistened and applied to the skin, the point at which they enter will become inflamed and an eruption will appear, which is commonly known to the barefoot boys and girls as "ground-itch." Moreover, by the end of eight weeks, examination and treatment will show the person to be harboring adult hook-worms, even though it may have been demonstrated that he was not infected up to the time he was brought in contact with the polluted soil. It should be remembered that the presence of "ground-itch" or "dew-itch," even though the skin may heal quickly, usually means that hookworms have entered the body and are beginning to rob the victim of his blood and vitality.

The second period in the life history of the hookworm begins with their entrance into the intestinal canal. There the male and female worms, after molting twice again, develop to adult life. From five to eight weeks is usually required for their growth. When grown they may usually be seen with the unaided eye. At this stage they fasten on the wall of the intestine, live for many years, and do the harm described next. Besides this the females deposit thousands of eggs, which, being mixed with the excreta, pass out on the ground to spread the disease.

Now that we have followed the complete life cycle of the parasites, let us study the disease caused by the adult worm in the intestine.

### The Effects of Hookworm Disease.

When we consider that several hundred hookworms may live in the intestine at one time, and that they may live there from six to ten years—sapping the blood, wounding the intestine, and poisoning the body—it is not strange that the body becomes diseased. The blood is needed to collect oxygen in the lungs and the food from the intestine, and distribute them over the body. In the disease much blood is lost. Some of it is taken into the bodies of the worms, and a great deal more is lost by bleeding from the small wounds made by the worms. In severe cases of the disease, the blood is reduced to one-fourth or one-sixth of what it should be. In such cases we find the victim's normal color replaced by a pale, sallow complexion; the lips are pale; the mucous surfaces generally are pale, and the skin is of a pale, yellowish hue. The eyes are listless, the pupils dilated and not very responsive to light; often they present a blank stare, fish-like in character. The hair is dry and scant, especially in the armpits. The face and the ankles are often swollen, anemic ulcers frequently appear on the legs, and the abdomen is prominent, giving rise to the term "pot-belly." The chest is flat, and the shoulder blades stand out prominently, suggesting "angel wings." When the disease occurs during the growing period there is a marked retardation in development; that is to say, a boy or girl may not be developed at 18 beyond what would be expected of one at 13 or 14 years of age. The appetite is often perverted, so that the sufferer has a craving for a particular kind of food, and often for certain substances not foods. For example, victims of the disease frequently crave clay, and for this reason are tered "dirt-eaters." Again, coffee-grounds or salt may be the substance desired.

The intestinal wall is considerably damaged by the worms, and becomes tender to pressure over the pit of the stomach. Where the worms bite, raw surfaces are left, so that it is easy for any germs, such as may cause typhoid fever or tuberculosis, to get into the body and set up a disease more violent in character, and more frequently fatal, than hookworm disease. There may be severe headaches, lassitude, dizziness and inability to sleep. The heart is poorly nourished by the impoverished blood, yet it is called on to do the work necessary to keep the body supplied with oxygen from the lungs and food from the digestive tract. As a result, the heat's action becomes labored, so that hookworm disease is frequently mistaken for heart-disease or Bright's disease. Most of these cases can easily be cured by getting rid of hookworms.

It must not be inferred that every person who is infected with hookworm suffers with all the symptoms mentioned. Much of the infection is so mild that the presence of the disease might not be suspected. In cases of medium severity one or more of the symptoms will be present, but the existence of the disease cannot be confirmed until the eggs of the worms are demonstrated in the excreta by microscopic examination.

The disease, however, even in mild cases, is a menace for two reasons: First, any infection exerts a handicapping influence on the victim. This has been shown among students who, though mildly infected, were underdeveloped in size and were backward in their studies; and, being below the standard, they were more subject to other diseases. Second, the persons mildly infected are carriers and distributors of the hookworm eggs, and may become responsible for the disease in a severer form in themselves and in other persons.

### How Hookworm Disease Spreads.

We have seen how each of the female hookworms living in the intestines deposits hundreds—often from twelve to fifteen hundred—of eggs daily; that these eggs do not hatch while they remain in the bowel, but after they have passed out on the soil, where there is moisture and warmth—but not too much—they hatch into larvae. Both the eggs and larvae are too small to be seen with the naked eye. The larvae remain invisible in size until they can get into the intestine, which they may reach by being swallowed with food or by entering the skin, usually of the feet, there producing "ground-itch" or "dew-itch," and then making a long journey through the body until the intestine is reached.

When infected persons go from place to place they spread the infection. It is thought that the negro slaves brought the disease to America from the West Coast of Africa. Likewise, the disease may be carried from one community to another. Suppose we have a school district at A where there is no infection, and the people are healthy and thrifty; and from this community the son and daughter of Mr. Jones visit the Smiths, in the distant community of B, where 75 per cent. of the residents have hookworm disease, and the soil is teeming with the larvae; that is to say, there is heavy soil pollution. Neither the Smith nor the Jones family pays much attention to the use of privies; indeed, there is much doubt if either has one; for out of 189,586 rural homes in the Southern states inspected by the Rockefeller Sanitary Commission, 95,988 were found to have no privies at all and 87,156 to have open privies which allowed wastes to spread freely through the soil. The sun is warm, and the Jones children go barefooted and contract "ground-itch." They eat strawberries and plums which have been on the ground. By the time their visit is over they have become infected with grown hookworms. Returning, they begin to pollute the soil at their home. The other members of the Jones family get the infection, and by the end of the summer they are all becoming pale and puny. The district school at A opens. The Jones children, though not in their usual health, go to school. The school has been equipped with many of the modern conveniences, but has not been provided with privies. The need of them was not really felt. The boys concealed themselves in woods and undergrowth to the east, and the girls to the west of the schoolhouse. The Jones children, not knowing they have a disease, of course, do not know they are spreading one when they use the common hiding-grounds. In ignorance of the diesase and in ignorance of sanitation, which the school should be teaching in a practical way, the soil around the school becomes heavily polluted, and a center for spreading the disease from one family to another. Soon the community has hookworm disease. Sometimes every pupil in a school gets it. The people becomes sick, backward, lazy, poverty-stricken and "trifling;" no one seems to suspect when, how or why "hard times" overtook the community. Had there been sanitary privies in use by all the people of the district, the disease would have been limited to the two Jones children, and their infection would have been mild. This is so, because when the hookworm eggs are collected in a sanitary privy they never reach the soil where, by hatching into larvae, they can do the harm.

### How the Disease Is Recognized.

Hookworm disease is recognized with certainty in two ways. If it is suspected from the clinical symptoms already described, the treatment, which is very simple and inexpensive, may be given. If the disease exists, the worms which cause it will be poisoned or dislodged by the medicine, and may be found in the stools. By washing the stools through one or two thicknesses of cheese-cloth, the worms will be left on the cloth, and may be seen and collected with ease.

The second method is the one commonly employed. Nearly a million persons have been examined in eleven Southern states by its use during the last four years. A small portion of the bowel movement is spread out thinly in water on a glass slide, and placed under a microscope. If the hookworm eggs, which are very characteristic in shape and size, are found, it is known that the hookworms are in the bowel, and that treatment for their removal should be taken. Hundreds of the best people, men, women and children in the South, are seeking examination by this method, and are putting aside all squeamishness in the determination to rid themselves and their communities of this pest.

### How Hookworm Disease Is Prevented.

There are two ways to get rid of hookworm disease, one is to prevent it, the other to cure it. Unfortunately as a people we are slow to apply preventive measures against disease. This means that we have too much disease, and, of course, that we rely too much on treatment. Preventive measures against hookworm disease are very simple. All that is necessary is to have the excreta of every person collected and disposed of in a way which will keep the hookworm eggs from developing. If this should be done in every case, the hookworms, not multiplying in the intestien, would die in a few years of old age and the disease would become extinct.

Until this can be accomplished much infection can be prevented by wearing shoes. The barefoot boy is a great gatherer of hookworms. But the really effective protection from the disease is to be found in clean soil. That can be secured when sanitary privies are constructed and regularly used by all the people. Even if those persons now infected should refuse treatment, but could be induced or required to use sanitary privies, the country would in a few years be freed from the disease.

The common pollution of the ground about schools and farmyards must be stopped. The tumble-down shack and surface privy which allow their contents to be spread over the soil, by rains, hogs and chickens, or carried by flies to the houses, must be abolished, and replaced everywhere by the sanitary privy, the essentials of which are that it have a water-tight, fly-screened receptacle, and that the contents be disposed of in a sanitary way, by burning, fermenting, or burying away from and below the water-supply. Human excrement should not be used as a fertilizer unless thoroughly treated under competent direction; otherwise it may carry hookworms to fruit and vegetables.

An inexpensive privy which can be easily provided for the most simple farm or cottage has water-tight pails, tightly closed in, close-fitting door, and all openings covered with wire mesh to exclude flies. Many communities are already putting in such privies, and there is a growing movement for placing them in every rural schoolyard of the South as a protection against hookworm, typhoid and other diseases.

All plans for privies approaching the ideal thus far presented seem to be too expensive or otherwise impractical. The feeling is growing that some practical arrangement should be recommended, even though from the point of the idealist it is not without danger. We know that trains, automobiles and elevators are dangerous to a degree, yet no one would be so foolish as to advise that we avoid the use of these conveniences of civilization until the element of danger is absolutely eliminated. Working on this principle, sanitarians are now recommending as a minimum for a privy, first that a hole be dug in the ground; second, that a substantial box with a hole in the bottom be turned upside down over the hole in the ground and the dirt banked around the lower edge of the box; third, that the hole in the box be covered when not in use; fourth, that the box be moved from time to time and the pit filled up with dirt. This privy may be built out in the bushes or it may be within expensively constructed walls. For all practical purposes an arrangement of this kind will eliminate hookworm eggs as a source of danger, and so long as it is fly-proof it will guard against the spread of typhoid fever by flies. If located a reasonable distance from the spring, or well, and below it, the danger of pollution is negligible, except perhaps in certain areas where the formation is largely limestone.

### How Hookworm Disease Is Cured.

Hookworm disease is usually treated with Epsom salts, and with powdered thymol given in capsules. The object of the Epsom salts is to free the intestine from mucus or other substances surrounding the hookworms and protecting them from the action of the thymol. The patient should take little or no supper on the evening before the thymol is to be administered. As early at night as is convenient he should take a dose of Epsom salt. The next morning as early as the salt has acted, half the number of capsules of thymol prescribed for the whole treatment should be taken. Two hours later the remaining capsules should be taken. Two hours after the second dose of thymol, another dose of Epsom salt should be taken, which will expel the hookworms that have been forced to loosen their hold on the intestinal wall by the action of the thymol, and will also get rid of the excess of thymol before it has had time to produce any harmful effects on the patient. Nothing should be eaten on the day the capsules are taken until the final dose of Epsom salt has acted well. A little water or strong coffee, WITHOUT milk, should alone be allowed.

As alcohol and oils dissolve thymol, making it actively poisonous to the patient, the use of them in any form would be exceedingly dangerous. Gravy, butter, milk, all alcoholic drinks and patent medicines, which generally contain alcohol, should be forbidden on the evening before and on the day of the treatment. Moreover, as many hookworm patients have dilated stomachs which do not readily empty themselves and it is important that the thymol reach the small intestine at once, the patient should lie on the right side for at least half an hour after taking each dose of thymol.

| Age, Years | Grains | Grams | 6 a. m. | 8 a. m. |
|---|---|---|---|---|
| 1 to 5 | 7.5 | .5 | ½ dose | ½ dose |
| 5 to 10 | 15. | 1. | ½ dose | ½ dose |
| 10 to 15 | 30. | 2. | ½ dose | ½ dose |
| 15 to 20 | 45. | 3. | ½ dose | ½ dose |
| 20 to 60 | 60. | 4. | ½ dose | ½ dose |
| 60 and upward | 45. | 3. | ½ dose | ½ dose |

The dose of thymol varies with the age of the patient. As the disease retards development and persons 18 years old often have only the normal growth of 13, apparent and not actual age determines the dose. A competent physician, of course, should supervise the treatment. The accepted scale of doses is shown by the accompanying table.

The thymol is powdered and given in capsules. If sugar of milk is added grain for grain, the thymol operates better.

In a majority of cases two treatments like the one just described will expel all the worms. In 1,518 out of 3,630 patients treated in Porto Rico, a single treatment effected a cure; a second treatment was sufficient in 1,166 cases; 518 required a third; 247 a fourth; 104 a fifth; 47 a sixth, and so on until the last case was freed from hookworms by the eleventh treatment. Frequently the worms not killed by the thymol are sickened to a degree that they do not deposit any eggs for approximately two weeks. By a microscopic examination, made two weeks or longer after the last treatment, it is possible to know when all of the worms are destroyed, and the treatment completed. When a microscopic examination is not possible, the feces expelled by each treatment can be examined for hookworms in the manner already described. When no more worms are seen, one extra treatment for good measure should be given.—Jour. Am. Med. Assn.

---

